# Potential Networks of Nitrogen-Phosphorus-Potassium Channels and Transporters in Arabidopsis Roots at a Single Cell Resolution

**DOI:** 10.3389/fpls.2021.689545

**Published:** 2021-06-16

**Authors:** Dhondup Lhamo, Sheng Luan

**Affiliations:** Department of Plant and Microbial Biology, University of California, Berkeley, Berkeley, CA, United States

**Keywords:** root single cell transcriptomics, expression analysis, nutrient transporters, nitrate transport, phosphate transport, potassium transport, NPK transport networks

## Abstract

Nitrogen (N), phosphorus (P), and potassium (K) are three major macronutrients essential for plant life. These nutrients are acquired and transported by several large families of transporters expressed in plant roots. However, it remains largely unknown how these transporters are distributed in different cell-types that work together to transfer the nutrients from the soil to different layers of root cells and eventually reach vasculature for massive flow. Using the single cell transcriptomics data from Arabidopsis roots, we profiled the transcriptional patterns of putative nutrient transporters in different root cell-types. Such analyses identified a number of uncharacterized NPK transporters expressed in the root epidermis to mediate NPK uptake and distribution to the adjacent cells. Some transport genes showed cortex- and endodermis-specific expression to direct the nutrient flow toward the vasculature. For long-distance transport, a variety of transporters were shown to express and potentially function in the xylem and phloem. In the context of subcellular distribution of mineral nutrients, the NPK transporters at subcellular compartments were often found to show ubiquitous expression patterns, which suggests function in house-keeping processes. Overall, these single cell transcriptomic analyses provide working models of nutrient transport from the epidermis across the cortex to the vasculature, which can be further tested experimentally in the future.

## Introduction

**FIGURE 1:**
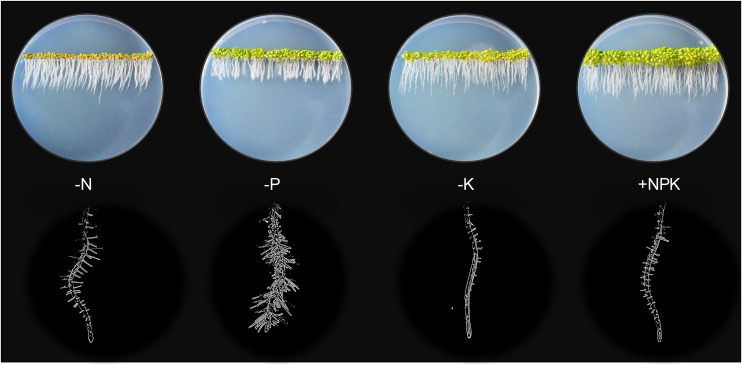
Plant growth highly depends on the continual nutrient supplies. Without N, P, or K (0 mM, 1/6 MS), 5-days old Arabidopsis seedlings displayed stunted shoot growth (top rows) and modified root architectures including primary root and root hair growth (bottom rows) compared to the sufficient-nutrient (1/6 MS) conditions. MS for Murashige and Skoog media.

Plant growth and development depend on the constant supply of mineral nutrients through the root system. Therefore, a deficiency in macronutrients such as NPK (Nitrogen, phosphorus and potassium) can have a profound impact on root growth and architecture (Figure 1), which in turn can alter the efficiency of nutrient acquisition and transport (López-Bucio et al., 2003; Gruber et al., 2013; Giehl and von Wiren, 2014; Luan et al., 2017). Plants possess large sets of nutrient transporters to perform these functions, among which many remain uncharacterized. Because the levels of these macronutrients in natural soils are often limiting plant growth, crop production often relies on heavy use of chemical fertilizers, which is not sustainable and pollutes the environment (Poirier and Bucher, 2002; Luan et al., 2017). To support sustainable agriculture, there is an urgent need to enhance their nutrient use efficiency (NUE), which will require decoding the complex genetic and biochemical components of nutrient transport.

Decades of research have advanced our understanding on the physiological roles of many nutrient transporters. One important aspect of transporter function is its site of activity. By combining genetic approaches with expression pattern analysis using promoter-reporter transgenic plant, studies can link the phenotype of a mutant to the tissue or cell-type where a particular transporter is expressed to make a functional prediction. This approach has identified a number of nutrient transporters that function in nutrient uptake in roots, root-to-shoot translocation, stomatal movement, pollen tube elongation and reproductive development (Wang and Wu, 2013; Gu et al., 2016; Wang et al., 2018). Although highly effective, this approach is time-consuming, labor-intensive, and limited by the number of genes and cells it can assess at a time. Recent breakthrough in single cell RNA-sequencing (scRNA-seq) provides a high-throughput platform to assess global gene expression at a single cell resolution, with the capacity to measure thousands of cells of different types and developmental stages simultaneously in a single experiment (Macosko et al., 2015; Ziegenhain et al., 2017). In the context of nutrient transport, scRNAseq can map the cell-type profiles of a large number of transporters so that we can predict where they may function and how they may be connected in a systematic process for nutrient handling at whole root or whole plant level.

The scRNAseq technology has been successfully applied to *Arabidopsis thaliana* roots for spatiotemporal characterization of distinct root cell-types, their developmental trajectories, and their transcriptional regulatory pathways (Denyer et al., 2019; Jean-Baptiste et al., 2019; Ryu et al., 2019; Shulse et al., 2019; Zhang et al., 2019). An Arabidopsis root atlas is generated by integrating two of the previously published datasets (Denyer et al., 2019; Ryu et al., 2019), and newly produced datasets from total of 110,427 single cells to profile gene expression dynamics in 11 different root cell-types (Shahan et al., 2020). Here, we leveraged this enormous dataset to map the cell-type expression pattern of NPK transporters in the Arabidopsis roots. Such analyses identified the putative transport map that allow nutrient transport longitudinally from the root cap (lateral root cap/LRC and columella) to the quiescent center (QC), and radially from the root epidermis (trichoblast and atrichoblast) to cortex and endodermis to reach stellar cells (pericycle, xylem, procambium and phloem) for root-to-shoot translocation. We first examined the expression patterns of known nutrient transporters in each family. This allowed us to gain more insights into the known NPK transporters at the root single cell resolution, which has not been fully achieved before. Then, we profile the expression patterns of uncharacterized nutrient transporter genes to predict their functional roles, considering their membrane localizations and phylogenetic relationship to the functionally characterized NPK transporters. However, this single cell profiling is currently limited to roots of young seedlings (5–7 days-old) under the normal growth conditions, which may not remain the same throughout the plant life cycle and under the changing nutrient status. For instance, 5-days-old seedlings respond to low-nutrient (N, P or K) stresses differently, with a different degree of modification to the root architectures and shoot growth (Figure 1). The transcriptional profiles of nutrient transporters under these stresses may be different compared to the controls and between the treatments. Therefore, low-nutrient-induced transporters may not be fully captured under the normal growth conditions presented in this study. However, these provide future opportunities to generate more detailed Arabidopsis root atlas that cover dynamic transcriptional changes of nutrient transporters over the course of plant development under changing nutrient status. This coupled with the physiological characterization of nutrient transporters will decipher the complexity of nutrient transport processes in plants. Our study identifies nutrient transporters that may function in collaborative fashion to distribute nutrients throughout the root system in concentric manners under non-stress conditions to support normal plant growth. In addition, it aims to lay the foundation for future research to dissect the complex networks of nutrient transport systems at a single cell resolution under varieties of biotic and abiotic stresses of different plant tissues and organs.

## Results and Discussion

### Nitrate Transport

Nitrate (NO_3_^–^) is the predominant form of N available in the soil and absorbed by plants for the synthesis of amino acids, nucleic acids, and many secondary metabolites (Noguero and Lacombe, 2016; Bellegarde et al., 2017; Fan et al., 2017). NO_3_^–^ not only serves as a major macronutrient but also acts as a signaling molecule that regulates plant developmental processes including seed germination, lateral root development, and flowering time (Bellegarde et al., 2017; Fan et al., 2017; Fredes et al., 2019). However, NO_3_^–^ is often limited in natural soils (<1 mM) and crop production relies on heavy fertilizer application that can reach up to 70 mM in agricultural lands, leading to runoff and pollution of aquatic ecological systems (Reisenauer, 1966; Krapp et al., 2014). To combat the constantly changing NO_3_^–^ status in the environment, plants have evolved large sets of NO_3_^–^ transporters for efficient uptake and distribution throughout plant cells and tissues (Krapp et al., 2014). These processes are mediated by four major NO_3_^–^ transporter families, which include NPF (Nitrate Transporter 1 (NRT1)/Peptide Transporter (PTR) Family), NRT2 (Nitrate Transporter 2), CLC (Chloride Channel), and SLAC/SLAH (Slow Anion Channel/SLAC1 homolog) (Krapp et al., 2014; Fan et al., 2017; Wang et al., 2018). While some of these NO_3_^–^ transporters in each family have been functionally characterized, the majority of them remains to be evaluated, especially those in the large NPF family. We examined the expression of these NO_3_^–^ transporters at a single cell resolution to understand how plants spatially distribute these transporters in the root system to charge the uptake, and transport of NO_3_^–^ from the external environment to the xylem vessels for long-distance translocation. The potential network we presented here of NO_3_^–^ channels and transporters is mainly applicable to young seedlings under the sufficient nutrient supply. It is solely based on the transcriptional profiles, which needs to be backed up by sufficient physiological studies in the future.

#### NPF Transporters

**FIGURE 2:**
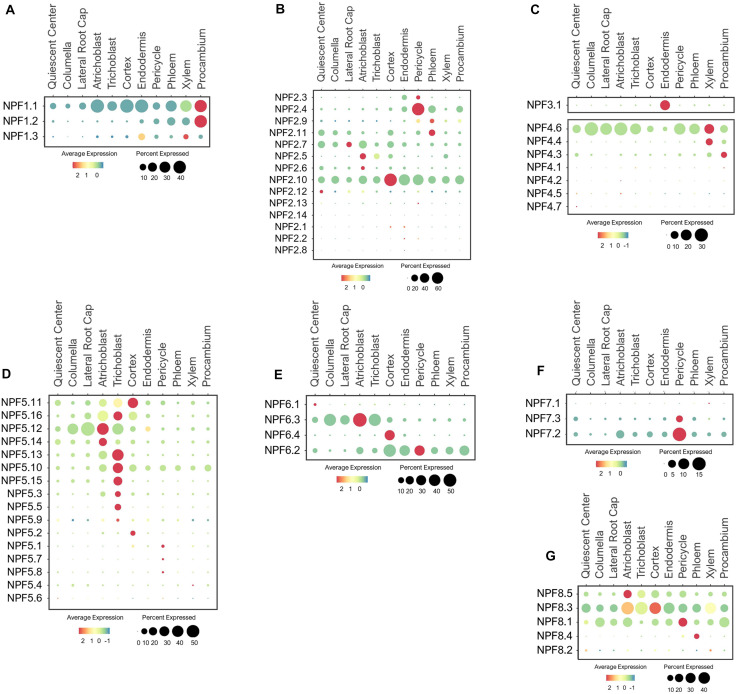
Expression of the low-affinity NPF transporters in Arabidopsis root single cells, **(A,B)** NPF1-2, **(C)** NPF3/4, and **(D–G)** NPF5-8. In dot plots, the circle size represents the proportion (%) of cells expressing a given gene, and the color of scale bar represents the mean expression (natural log +1 pseudocount).

The Arabidopsis NPF family includes 53 members that display high sequence homology with the SLC15/PepT/PTR/POT family of peptide transporters in animals (Léran et al., 2014; O’Brien et al., 2016; Wang et al., 2018). NPF transporters are mostly located at the plasma membrane (PM) and serve as low-affinity transporters that transport NO_3_^–^ and other diverse metabolites and hormones (Krapp et al., 2014; Léran et al., 2014; Wang et al., 2018). NPFs are divided into eight subfamilies based on their phylogenetic relationships (Léran et al., 2014). NPF1 contains three members, among which the PM-localized NPF1.1 (NRT1.12) and NPF1.2 (NRT1.11) were involved in transferring root-derived NO_3_^–^ into phloem of mature leaves for redistribution to young leaves (Hsu and Tsay, 2013). Root single cell transcriptomes indicated these NPF1s were broadly expressed in stellar cells, with the highest expression in the procambium (Figure 2A), the meristematic tissue that give rise to xylem and phloem (Jouannet et al., 2015; De Rybel et al., 2016). This expression pattern suggests NPF1.1/1.2 may function in NO_3_^–^ loading to both meristematic and mature phloem and xylem cells in developing roots under the normal growth conditions. The function of NPF1.3 has not been previously characterized. Based on its single cell profile, NPF1.3 was expressed at a much lower level as compared to NPF1.1/1.2, mainly in endodermal and xylem cells (Figure 2A), possibly to translocate NO_3_^–^ to shoots. Its expression may amplify during the later stage of development or under a specific nutrient stress condition. The broad expression of NPF1.1 in other cell-types except root cap suggests its potential contribution to the radial transport and distribution of NO_3_^–^ to or from stellar cells.

The NPF2 subfamily contains 14 members, several of which have been functionally characterized. For example, the PM-localized NPF2.3 (NAXT2) mediates NO_3_^–^ efflux from pericycle to xylem for root-to-shoot transport in response to salt stress (Taochy et al., 2015). As expected, NPF2.3 was mainly expressed in the pericycle, but at a low level under the normal conditions (Figure 2B), suggesting it may be more stress-responsive. NPF2.4 was reported to specifically mediate chloride (Cl^–^) loading to xylem in response to salt stress (Li et al., 2016). Consistent with this function, NPF2.4 was expressed in the pericycle (Figure 2B), but its high expression under the normal growth conditions suggests it may also mediate Cl^–^ transport under the non-stress conditions. NPF2.9 (NRT1.9) was shown to recirculate NO_3_^–^ from aerial tissues to roots via phloem loading (Wang and Tsay, 2011). As expected, NPF2.9 was expressed in phloem at a low level (Figure 2B), implying NO_3_^–^ recirculation may not be a high priority under the nutrient sufficiency. NPF2.11 (GTR2) can transport NO_3_^–^/gibberellin/glucosinolate, and mediated source-to-sink transport of glucosinolate via phloem loading (Nour-Eldin et al., 2012; Andersen et al., 2013; Saito et al., 2015; Wang et al., 2018). Consistent with these studies, NPF2.11 was expressed in phloem (Figure 2B). Its function may overlap with NPF2.9 in NO_3_^–^ loading to phloem based on their similar expression patterns. NPF2.7 (NAXT1) extrudes NO_3_^–^ from root cells under the acidic environments (Segonzac et al., 2007). In compliance with its physiology, NPF2.7 was expressed in the cortex, QC, epidermis, and root cap (Figure 2B). NPF2.5/2.6 are closely related to NPF2.7, and may share similar functions in NO_3_^–^ secretion from root epidermis to maintain ionic balance at the root-soil interface. A previous study reported the involvement of NPF2.5 in Cl^–^ extrusion from roots under salt stress (Li et al., 2017a), however, its NO_3_^–^ transport activity needs further evaluation. Among all the NPF2s, NPF2.10 displayed a ubiquitous expression, signifying its potential function in NO_3_^–^ distribution across different root cell layers. Studies have shown that NPF2.13 (NRT1.7) recycles NO_3_^–^ from old to developing leaves by directly loading NO_3_^–^ to the phloem (Fan et al., 2009), and NPF2.12 (NRT1.6) delivers NO_3_^–^ from source leaves to developing seeds (Almagro et al., 2008). As a result of specific expression and function in leaf and seed developments, these two transporters were barely expressed in roots (Figure 2B).

As the only member in the NPF3 clade, NPF3.1 was reported to be a PM-localized transporter involved in nitrite and NO_3_^–^ transport in leaves, and GA transport to root endodermis (Sugiura et al., 2007; Pike et al., 2014; Tal et al., 2016). Consistently, it exhibited endodermis-specific expression in the developing roots under the normal conditions (Figure 2C). This suggests that NPF3.1 may enable NO_3_^–^/nitrite/GA transport across the casparian strip barrier.

Seven NPF4 members are present in Arabidopsis, among which, only NPF4;6 (NRT1.2/AIT1) has been characterized to function as a low-affinity NO_3_^–^ uptake transporter (Huang et al., 1999). As anticipated, NPF4.6 was expressed in epidermis and root tip (QC, columella and LRC) to initiate NO_3_^–^ acquisition (Figure 2C). In addition, its high expression in stellar cells, especially xylem, suggests a potential role in NO_3_^–^ loading to xylem. We hypothesize that NPF4.4 may also be involved in xylem loading because its expression was exclusively associated with xylem. NPF4.3 was preferentially expressed in procambium, suggesting its potential role in xylem and/or phloem loading. Their potential functions in long-distance transport needs to be evaluated experimentally.

While the majority of NPFs are found at the PM, NPF5.11/5.12/5.16, among the sixteen NPF5 members, display the tonoplast localization to remobilize NO_3_^–^ from the vacuoles of stellar cells, especially pericycle (He et al., 2017). However, these transporters were mainly expressed in root surface and cortical cells based on the single cell profiles of young seedlings under the normal growth conditions (Figure 2D), necessitating a more detailed examination of their function in the future. Intriguingly, the other NPF5s were also largely expressed in the epidermis, especially trichoblast, suggesting a role in soil-root exchange. Because the subcellular locations of these NPF5s remain to be determined, there is a gap of knowledge regarding their physiological functions. The single cell expression patterns here provide a reference point for functional analysis using genetic procedures. Among the NPF5 subfamily, NPF5.10 was expressed more broadly in stellar cells, and NPF5.1/5.7/5.8 specifically in a few pericycle cells. Depending on their subcellular locations and transport activities, these transporters could serve as candidates for NO_3_^–^ transfer or remobilization from stellar cells to xylem vessels for long-distance transport. In addition, some of their expressions may be more responsive to nutrient stresses or during later-stages of the plant development.

NPF6 has four members, among which, NPF6.3 (NRT1.1/CHL1) functions as a dual-affinity transporter that mediate NO_3_^–^ uptake and nitrate-dependent auxin transport (Huang et al., 1996; Wang et al., 1998; Liu et al., 1999; Krouk et al., 2010). As the key transporter in NO_3_^–^ acquisition, NPF6.3 was highly expressed in epidermis and root cap (Figure 2E). Root cap cells, located at the root tip are essential for nutrient sensing and uptake, root penetration in soils, root growth, gravitropic and hydrotropic responses, which are mostly controlled by polar auxin transport (Svistoonoff et al., 2007; Arnaud et al., 2010; Ruiz Herrera et al., 2015). Studies have shown NPF6.3 at the root tip is able to sense the changes in soil NO_3_^–^ status, and transduces NO_3_^–^ signaling to regulate nitrate uptake and auxin distribution for lateral root development (Liu and Tsay, 2003; Ho et al., 2009; Krouk et al., 2010; Gojon et al., 2011; Mounier et al., 2014; O’Brien et al., 2016). Other members in the same subfamily displayed divergent expression patterns. For example, NPF6.4 (NRT1.3) showed cortex-specific expression while NPF6.2 (NRT1.4) was broadly expressed in cortical and stellar cells. The more distant member, NPF6.1 expression was not detected in roots under the conditions provided. These NPF6s may have evolved to perform distinct functions in transporting NO_3_^–^ radially from root surfaces to inner vasculature.

Among the three NPF7 transporters, the closely related NPF7.3 (NRT1.5) and NPF7.2 (NRT1.8) mediate opposite functions in long-distance transport. NPF7.3 has been shown to export NO_3_^–^ and K^+^ from pericycle to xylem for shoot translocation, whereas NPF7.2 retrieves NO_3_^–^ from xylem and retain NO_3_^–^ in root cells (Lin et al., 2008; Li et al., 2010, 2017b; Drechsler et al., 2015). Interestingly, these two homologs shared high expression in the pericycle (Figure 2F), suggesting that their transport mode may be opposite, one mediating influx and one efflux in the same cell-type.

The five NPF8 proteins are not well understood concerning their NO_3_^–^ transport properties. NPF8.1/8.2/8.3 (PTR1/5/2) are shown to transport dipeptide, hormones and/or amino acid (Corratgé-Faillie and Lacombe, 2017). NPF8.2 is preferentially expressed in flowers, where it transported dipeptide across the PM of germinating pollen (Komarova et al., 2008; Hammes et al., 2010). Thus, its expression was barely detected in roots (Figure 2G). The tissue- and cell-specific expression for other NPF8 transporters have not been previously reported. In this study, we found that these NPF8.1/8.3/8.5 were broadly expressed in multiple root cell-types. If these transporters are localized to the PM and capable of transporting NO_3_^–^, they may function with NPF6.3 and NPF4.6 in NO_3_^–^ uptake at the root surfaces. In addition, NPF8.1/8.3 were also expressed in stellar cells, suggesting their involvement in long-distance transport. NPF8.4 may have more specific function in NO_3_^–^ loading to phloem based on its phloem-specific expression. While these expression patterns provide a valuable information regarding their potential functions, analyses of their subcellular localizations, transport properties and physiological roles are necessary for validations.

#### NRT2 Transporters

**FIGURE 3:**
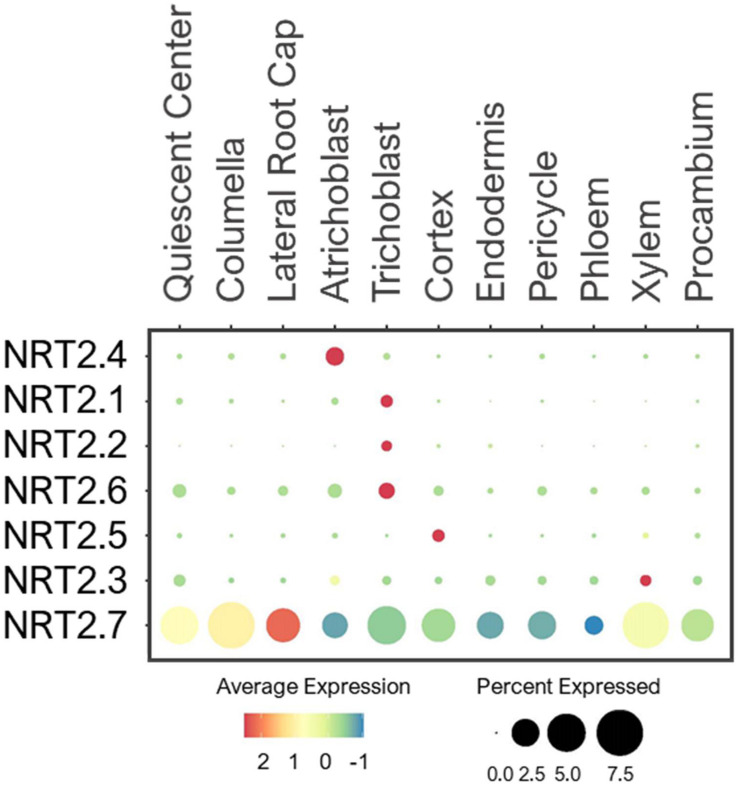
Expression of the high-affinity NRT transporters in Arabidopsis root single cells. In dot plot, the circle size represents the proportion (%) of cells expressing a given gene, and the color of scale bar represents the mean expression (natural log +1 pseudocount).

Unlike NPF, the NRT2 family comprises seven proteins that are considered high-affinity NO_3_^–^ transporters (Orsel et al., 2002; Krapp et al., 2014). These proteins were identified by sequence homology to NRT2.1, which was isolated by a differential display approach due to highly NO_3_^–^inducible expression (Filleur and Daniel-Vedele, 1999; Orsel et al., 2002). NRT2.1/2.2/2.4/2.5 were shown to be PM-localized transporters, involved in NO_3_^–^ uptake especially under low-NO_3_^–^ stress (Cerezo et al., 2001; Filleur et al., 2001; Kiba et al., 2012; Lezhneva et al., 2014). Because their expressions are highly induced by low-NO_3_^–^, the single cell profiling detected only low levels of expression in roots under the normal conditions. Nevertheless, they were expressed in root epidermis (Figure 3), consistent with their uptake functions. NRT2.7 is the only transporter located at the tonoplast, where it is shown to import NO_3_^–^ into vacuoles of seeds (Chopin et al., 2007). However, it was also broadly expressed in various cell-types in young roots compared to the other NRT2s (Figure 3). This suggests that NRT2.7 may serve as a housekeeping function to sequester excess NO_3_^–^ into the vacuole in multiple tissues.

#### CLC Antiporters

**FIGURE 4:**
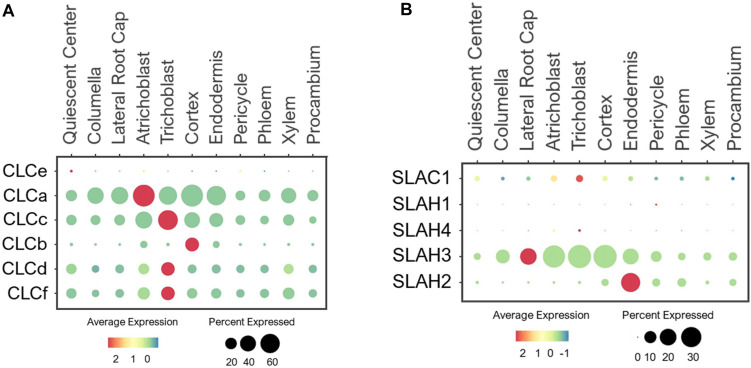
Expression of the **(A)** CLC antiporters and **(B)** SLAC1 channels in Arabidopsis root single cells. In dot plots, the circle size represents the proportion (%) of cells expressing a given gene, and the color of scale bar represents the mean expression (natural log +1 pseudocount). The expression of CLCg was not detected in any single cells.

Arabidopsis CLCs were identified by the sequence homology to voltage-gated chloride channels in animals (Hechenberger et al., 1996; Lv et al., 2009). The plant CLC family contains both channels and antiporters that transport anions including NO_3_^–^ (Fan et al., 2017). The Arabidopsis genome encodes 7 CLC members. CLCa-c, and CLCg were localized to tonoplast, CLCd/f to Golgi, and CLCe to chloroplast (Fecht-Bartenbach et al., 2007; Marmagne et al., 2007; Lv et al., 2009; Barbier-Brygoo et al., 2011). Because CLCe is shown to be specifically expressed in green tissues (Monachello et al., 2009), its expression in roots was undetected (Figure 4A). CLCa is a major component that drives NO_3_^–^ accumulation into vacuoles (Geelen et al., 2000; De Angeli et al., 2006). Its functional significance was evident from the ubiquitous expression in all cell-types in developing roots under the normal conditions (Figure 4A). Likewise, CLCc was widely expressed, thus may share similar tasks as CLCa in vacuolar NO_3_^–^ sequestration. The expression of CLCb was specifically detected in the cortex, suggesting a specific function of this CLC in storing NO_3_^–^ in the vacuole of cortical cells. Additionally, two Golgi-localized transporters, CLCd and -f showed ubiquitous expression in all cell-types, implicating them in the housekeeping function of anion distribution in Golgi apparatus of all cell-types.

#### SLAC Channels

SLAC1 shares homology to TehA transporters in bacteria, archaea and fungi (Chen et al., 2010; Dreyer et al., 2012). SLAC1 and its four homologs, SLAH1-4, are anion channels that conduct NO_3_^–^ and Cl^–^ transport across the PM (Negi et al., 2008; Barbier-Brygoo et al., 2011; Fan et al., 2017). SLAC1 and SLAH3 were shown to mediate NO_3_^–^/Cl^–^ efflux and regulate stomatal closure (Schmidt and Schroeder, 1994; Vahisalu et al., 2008; Geiger et al., 2009b, 2011; Chen et al., 2010; Zheng et al., 2015). Because SLAC1 is preferentially expressed in guard cells (Negi et al., 2008), it was not detected in roots (Figure 4B). SLAH1/3 are earlier reported to be expressed in root pericycle to mediate Cl^–^ efflux to xylem (Cubero-Font et al., 2016). SLAH2 is shown to be a NO_3_^–^-specific channel that may facilitate root-to-shoot NO_3_^–^ transport (Maierhofer et al., 2014). Based on their expressions in stellar cells, especially pericycle, SLAH1-3 have been speculated to drive NO_3_^–^ loading to xylem (Hedrich and Geiger, 2017). We examined their expressions based on single cell profiles and found that the closely related SLAH1/4 were barely detected in the developing roots under the normal conditions (Figure 4B). These genes may require specific growth conditions or plant developmental stages for induction. On the other hand, SLAH2/3 showed distinct expression patterns. While SLAH3 was broadly expressed in epidermis, root cap, cortical and endodermal cells, SLAH2 displayed predominant expression in endodermis (Figure 4B), suggesting an endodermis-specific function for SLAH2. The broad expression pattern of SLAH3 is consistent with its functions in diverse processes (Gutermuth et al., 2013; Zheng et al., 2015; Cubero-Font et al., 2016; Zhang et al., 2016; Hedrich and Geiger, 2017).

#### Potential Network of Nitrate Channels and Transporters in Roots

**FIGURE 5:**
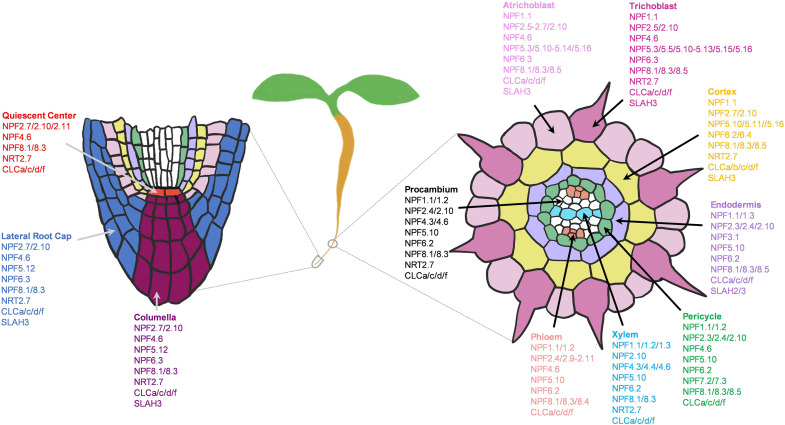
Potential network of nitrate (NO_3_^–^) channels and transporters in the root system of 5–7 days old Arabidopsis seedlings grown under the normal growth conditions. Expressed NO_3_^–^ transporters involved in longitudinal transport (left) at the meristematic zone and radial transport (right) at the elongation/maturation zones were shown. Nitrate transporter genes not expressed under the normal growth conditions were excluded.

In Arabidopsis, 73 NO_3_^–^ transporters belonging to the NPF, NRT2, CLC, and SLAC1/SLAH families are identified, among which, only several have been characterized to function as NO_3_^–^ transporters in roots (Krapp et al., 2014; Wang et al., 2018). Through single cell transcriptomic analysis, we were able to expand this list, and map the potential network of NO_3_^–^ channels and transporters in the root system of young seedlings under the normal growth conditions (Figure 5). The previous studies reported the roles of NPF4.6/6.3 and NRT2.1/2.2/2.4/2.5 in NO_3_^–^ uptake from root surface cells (Huang et al., 1996, 1999; Wang et al., 1998; Liu et al., 1999; Cerezo et al., 2001; Filleur et al., 2001; Kiba et al., 2012). We additionally found several NPF2/5/8, CLCs, NRT2.7 and SLAH3 in the root tip and epidermis, where they might participate in NO_3_^–^ uptake, efflux or storage/distribution to adjacent cells (Figure 5). Many of these transporters were also expressed in the cortex and endodermis to distribute NO_3_^–^ toward stellar cells. Among these families, the putative PM-localized NPF2/4/6/8 members are likely involved in NO_3_^–^ uptake, transfer and long-distance transport; SLAH3 in NO_3_^–^ distribution; CLCs in NO_3_^–^ sequestration into vacuoles (also NRT2.7) and Golgi; and the putative vacuolar NPF5s in NO_3_^–^ remobilization.

In addition, we also found few transporters with cell-type specific expressions such as NPF6.4 and CLCb in cortex, and NPF3.1 and SLAH2 in endodermis. These cortex-specific transporters may be the major contributors to the higher NO_3_^–^ concentrations observed in the cortical cells compared to the epidermal cells, as reported in the previous study using the NO_3_^–^-selective microelectrodes (Zhen et al., 1991). Such single cell profiling of nitrate concentrations in NO_3_^–^ transporter mutants compared to the wild type may determine the function of a nutrient transporter in a specific cell type.

NPF2.3/7.3 was previously reported to export NO_3_^–^ from pericycle to xylem (Lin et al., 2008; Taochy et al., 2015; Li et al., 2017b), NPF7.2 in NO_3_^–^ retrieval from xylem (Lin et al., 2008; Li et al., 2010, 2017b; Drechsler et al., 2015), and NPF2.9 in NO_3_^–^ loading to phloem (Wang and Tsay, 2011). We additionally found several NPF1/2/4/5/6/8, NRT2.7 and CLC transporters expressed in stellar cells that might participate in long-distance NO_3_^–^ transport and distribution (Figure 5). Among these, several showed cell-type specific expressions, such as NPF5.1/5.7/5.8 in pericycle, NPF4.3 in procambium, NPF4.4 xylem, and NPF8.4 in phloem. These transcriptional profiles provide functional predictions of these NO_3_^–^ transporters in specific cell-types of small root tissues, grown under the normal conditions. More in-depth analysis of these genes under diverse nutritional and developmental conditions are required to reflect their main sites of action. Also, it is critical to determine the subcellular localizations and transport activities of these uncharacterized transporters to understand their mode of transport for NO_3_^–^ distribution. So far, the majority of the functionally known NO_3_^–^ transporters were PM-localized, with few found at the tonoplast and Golgi membranes. There still exists numerous novel NO_3_^–^ transporters that might localize to different subcellular compartments such as plastids, mitochondria, and ER in addition to vacuole and Golgi.

### Phosphate Transport

P is absorbed by plants in the form of inorganic phosphate (Pi) that serves as an integral component of nucleic acids, phospholipids and ATP, and participates in energy metabolism and signaling transduction (Poirier and Bucher, 2002; Chiou and Lin, 2011). Plants require 60–80 μM of cytosolic Pi to sustain growth, but the typical Pi concentration in the natural soil is extremely low (<10 μM) (Pratt et al., 2009; Plaxton and Tran, 2011; Luan et al., 2017). This is largely compensated by the intensive use of P-rock fertilizers, compromising the sustainability of natural resources and negatively impacting the environment (Chiou and Lin, 2011; López-Arredondo et al., 2014; Luan et al., 2017). In response to the changing Pi status in soils, plants have developed large families of Pi transporters for efficient Pi acquisition, translocation, storage and distribution throughout cells and tissues (Gu et al., 2016; Luan et al., 2017). These include five families of Phosphate Transporters (PHT1-5), Glycerol 3-phosphate permease (G3Pp) and Phosphate1 (PHO1) (Stefanovic et al., 2007; Gu et al., 2016; Xu et al., 2019; Lhamo et al., 2020). The membrane localizations of many of these Pi transporters have been elucidated, allowing us to better predict their functions in the root system using single cell transcriptomic datasets. This *in silico* approach has provided us the opportunity to create a potential network of Pi transporters that together direct the Pi flow from the epidermis to the vasculature. However, this is purely based on transcriptional profiling of Pi transporters at a single time-point (5–7 days old seedlings) and one condition (normal). While the present study serves as a starting point, the responses of Pi transporters during different plant life stages and stress conditions need to be evaluated in more details, backed up by sufficient genetic and physiological studies to support the putative model.

#### PHT1 Transporters

**FIGURE 6:**
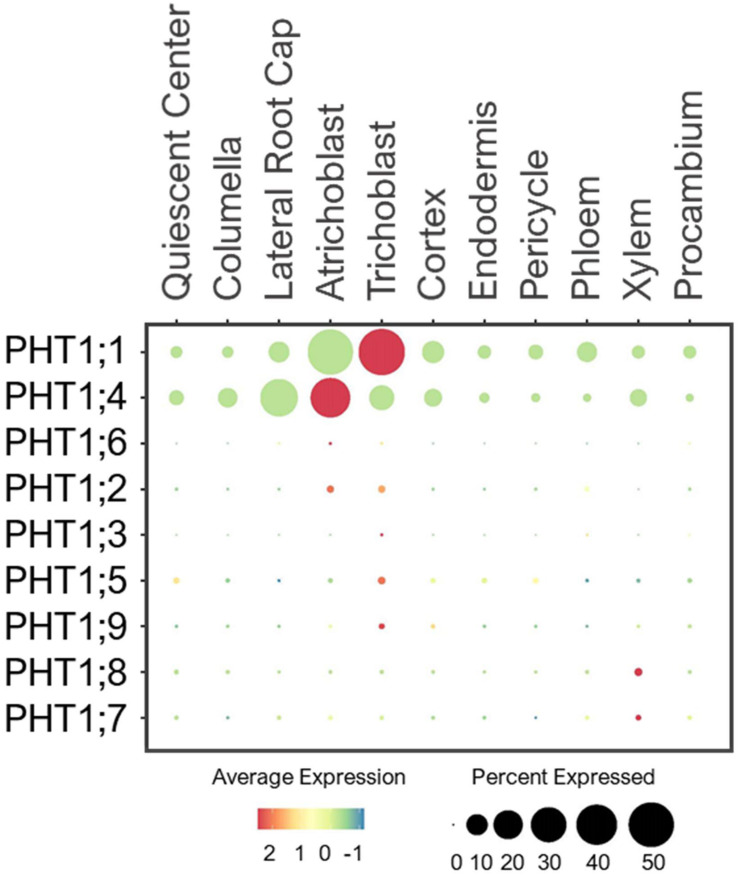
Expression of the high-affinity PHT1 transporters in Arabidopsis root single cells. In dot plot, the circle size represents the proportion (%) of cells expressing a given gene, and the color of scale bar represents the mean expression (natural log +1 pseudocount).

The PHT1 family contains nine members that share sequence homology to the yeast high-affinity H^+^/Pi symporter, PHO84 (Poirier and Bucher, 2002; Nussaume, 2011). PHT1s are located at the PM of root surface cells to mediate Pi uptake and translocation (Mudge et al., 2002; Nussaume, 2011). PHT1;1 and PHT1;4 are the dominant players in Pi absorption under a wide-range of Pi concentrations (Misson et al., 2004; Shin et al., 2004). Consistently, single cell profiles showed the high expression of these two transporters in root epidermis under the normal conditions (Figure 6). PHT1;4 was additionally expressed in LRC. This cell-type is shown to be important for sensing low-Pi stress to promote root growth arrest (Svistoonoff et al., 2007), as observed in Figure 1. In addition, a study using high-resolution real-time ^33^P imaging indicated that the root cap cells contribute 20% of the total seedling Pi uptake (Kanno et al., 2016). These suggest the role of PHT1;4 in Pi sensing and uptake at the root tip. This together with PHT1;1 at the epidermis makeup the largest portion of absorbed Pi in Arabidopsis seedlings (Shin et al., 2004).

PHT1;8 and PHT1;9 were shown to mediate Pi uptake from roots and translocation to shoots especially under low-Pi (Remy et al., 2012; Lapis-Gaza et al., 2014). In line with these studies, the single cell profiles revealed a low expression of PHT1;9 in epidermis and PHT1;8 in xylem of developing roots under the normal conditions (Figure 6). PHT1;7 was also lowly detected in xylem, and may share overlapping functions with PHT1;8 in translocating Pi from root to shoot. Because no definitive Pi transporters at the PM of stellar cells have been reported, we speculate that some of the PHT1 members such as PHT1;7/1;8 may be present to promote xylem loading, and their expression may be more responsive to low-Pi than the normal conditions provided in this study.

Other PHT1 members were lowly expressed in epidermis of young seedlings under the normal conditions (Figure 6), and might be more responsive to Pi starvation to initiate Pi uptake (Mudge et al., 2002; Nussaume, 2011). In addition to the condition requirement, some may function in later stages of plant development. For instance, PHT1;5 was shown to recycle Pi from source-to-sink (senescing leaves to young leaves and roots) in response to low-Pi (Mudge et al., 2002; Nagarajan et al., 2011; Nussaume, 2011). These indicate plants may not need to activate the transcription of all PHT1 members during the early growth stage when the Pi supply is sufficient.

#### PHT2/3/4 Transporters

**FIGURE 7:**
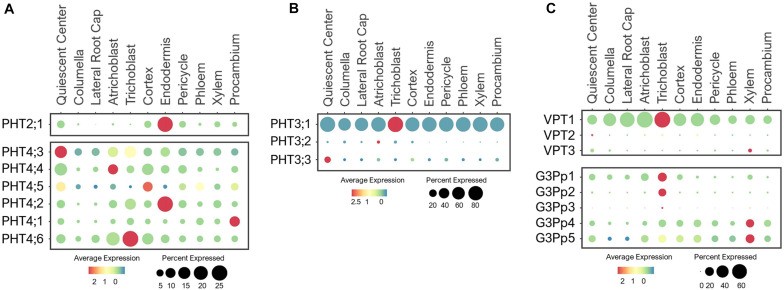
Expression of the **(A)** PHT2/4, **(B)** PHT3 and **(C)** VPT and G3Pp transporters in Arabidopsis root single cells. In dot plots, the circle size represents the proportion (%) of cells expressing a given gene, and the color of scale bar represents the mean expression (natural log +1 pseudocount).

PHT2/3/4/5 family transporters are located at different subcellular compartments where they participate in Pi storage and distribution throughout the plant (Gu et al., 2016; Luan et al., 2017). Several of these transporters have been characterized and shown to have function in green tissues. However, their expression and physiology in the root system are not well understood. For example, PHT2;1, a single member of the PHT2 family was shown to transport Pi into chloroplasts in leaves (Daram et al., 1999; Versaw and Harrison, 2002; Rausch et al., 2004). We detected PHT2;1 expression exclusively in root endodermis (Figure 7A), raising a question about its intriguing function in root Pi transport. We hypothesize that PHT2;1 in endodermis may contribute to Pi translocation to shoots, where its function is more prominent.

Similar to PHT2;1, five PHT4 members (PHT4;1–4;5) are targeted to plastids, and one (PHT4;6) in Golgi (Guo et al., 2008a, b). These PHT4s were shown to mediate Pi transport when expressed in yeast Pi transport mutants (Guo et al., 2008b). PHT4;1, PHT4;4, and PHT4;5 were reported to be expressed in leaves, PHT4;2 exclusively in roots, and PHT4;3 and PHT4;6 in both tissues (Guo et al., 2008a, b). Among these, PHT4;1 was shown to compartmentalize Pi in chloroplasts for ATP synthesis and photosynthesis (Guo et al., 2008a; Karlsson et al., 2015). PHT4;4 was reported to transport ascorbate into chloroplasts, and capable of transporting both Pi and ascorbate in liposomes (Miyaji et al., 2015). PHT4;2 was shown to perform reversible Na^+^-dependent Pi transport in root plastids (Irigoyen et al., 2011). Expression analysis of these PHT4 members in root single cells revealed broad expression in multiple cell-types of young roots under the normal growth conditions (Figure 7A), likely to support their housekeeping functions in plastids. However, these transporters had distinct preferential expressions. For example, PHT4;3 and PHT4;5 were highly expressed in QC and PHT4;1 in procambium, suggesting their roles in balancing plastid Pi in meristematic cells of the root tip and the vasculature, respectively. Also, PHT4;3/4;4/4;6 were highly expressed in epidermis, PHT4;5 in cortex, and PHT4;2 in endodermis. These PHT4s may coordinate with each other to control Pi fluxes in plastids throughout the root system, which is essential for the synthesis and distribution of amino acids, fatty acids and secondary compounds (Flügge et al., 2003). PHT4;6 is the only PHT4 member localized to the Golgi, where it was shown to mediate Pi efflux from Golgi to cytosol (Cubero et al., 2009; Hassler et al., 2012, 2016). In a single cell profile of developing roots, PHT4;6 was largely expressed in epidermis and cortex (Figure 7A). This suggests a potential function of PHT4;6 in maintaining Pi homeostasis in Golgi of these transit cell-types. Currently, the function of many PHT4 members in Pi transport and distribution in the root system and whether these may participate in long-distance transport are vaguely understood. We provided assumptions based on the known subcellular localization and expression profiling of these Pi transporters at one time point and growth condition. Their functions in the root system need to be verified experimentally.

The PHT3 family contains three members in Arabidopsis that show sequence homology to the first mitochondrial Pi translocator (MPT1) cloned from birch plants and yeast MIR1 (Kiiskinen et al., 1997; Takabatake et al., 1999; Hamel et al., 2004). These PHT3s transport Pi from cytosol into the mitochondrial matrix to support ATP synthesis and redox homeostasis (Zhu et al., 2012; Jia et al., 2015). PHT3;1 (MPT3) was reported to be ubiquitously expressed in multiple tissues, while PHT3;2 (MPT2), and PHT3;3 (MPT1) were only expressed in the green and/or reproductive tissues (Zhu et al., 2012). Among these, PHT3;1 had the largest contribution to the mitochondrial Pi homeostasis, which was shown to be indispensable for plant development (Jia et al., 2015). Consistent with these studies, PHT3;1 displayed ubiquitous expression in all root cell-types, while the other two were barely detected in developing roots under the normal growth conditions (Figure 7B). This emphasizes the essential role of PHT3;1 in supporting mitochondrial functions throughout the root system. Further studies of these mitochondrial transporters have been compromised by the lethality of their homozygous mutants (Jia et al., 2015).

#### VPT and G3Pp Transporters

In Arabidopsis, three members of the PHT5 family are responsible for Pi storage into vacuoles (Liu et al., 2015, 2016). VPT1 (PHT5;1) is the major player in vacuolar Pi sequestration (Liu et al., 2015, 2016; Luan et al., 2019). Single cell profiling revealed a ubiquitous expression of VPT1 in all root cell-types, while VPT2 (PHT5;2) and VPT3 (PHT5;3) were expressed at low levels in roots of young seedlings under the normal growth conditions (Figure 7C). This is consistent with the earlier functional analysis (Liu et al., 2015, 2016). However, the expressions of VPT2/3 may become more noticeable in roots of later stages or in response to a specific stress, such as Pi toxicity. In addition, these VPTs may function in other tissues or organs. For example, VPT3 along with VPT1 are shown to play role in the later stage of plant development for systemic allocation of Pi between leaves and flowers (Luan et al., 2019).

The stored Pi in vacuoles is remobilized by the G3Pp family transporters (Xu et al., 2019). This family contains five G3Pp members in Arabidopsis, among which, G3Pp1-3 (VPE1-3) are localized to tonoplast (Ramaiah et al., 2011; Luan and Lan, 2019; Xu et al., 2019). Only G3Pp2 has been confirmed to be a functional vacuolar Pi exporter (Xu et al., 2019). G3Pp4 is localized to the plastid envelope and mediated lipid accumulation in seeds (Kawai et al., 2014). The membrane localization of G3Pp5 remains uncharacterized, so are the functions of the majority of G3Pps. Single cell profiling of young seedlings under the nutrient sufficiency displayed G3Pp1 expression in root tip, epidermis and cortex, and G3Pp2 exclusively in trichoblast, whereas G3Pp3 was barely detected (Figure 7C). Considering these G3Pps as efflux transporters, G3Pp1/2 may work concurrently with VPT1 in controlling Pi fluxes between vacuoles and cytosol of epidermal cells to maintain stable cytosolic Pi concentration, and might aid in Pi distribution to the adjacent cells under the normal conditions. Their roles in vacuolar Pi remobilization are critical for low-Pi adaptation, and as such, these genes were shown to be highly induced by low-Pi stress (Ramaiah et al., 2011). On the other hand, the G3Pp3 expression was highly upregulated under various low-nutrient (Pi, K^+^, NO_3_^–^, and Iron) stresses (Ramaiah et al., 2011), suggesting the specific role of this transporter in stress-response. Based on their putative functions, we anticipate a wider expression of these G3Pps in different root cell-types upon nutrient-stress conditions. G3Pp4 and its close homolog, G3Pp5, shared broad expression in multiple cell-types including xylem during normal growth conditions (Figure 7C). These G3Pps may contribute to Pi distribution via Pi efflux from plastids, however, their Pi transport mechanisms and physiological functions need to be confirmed.

#### PHO1 Transporters

**FIGURE 8:**
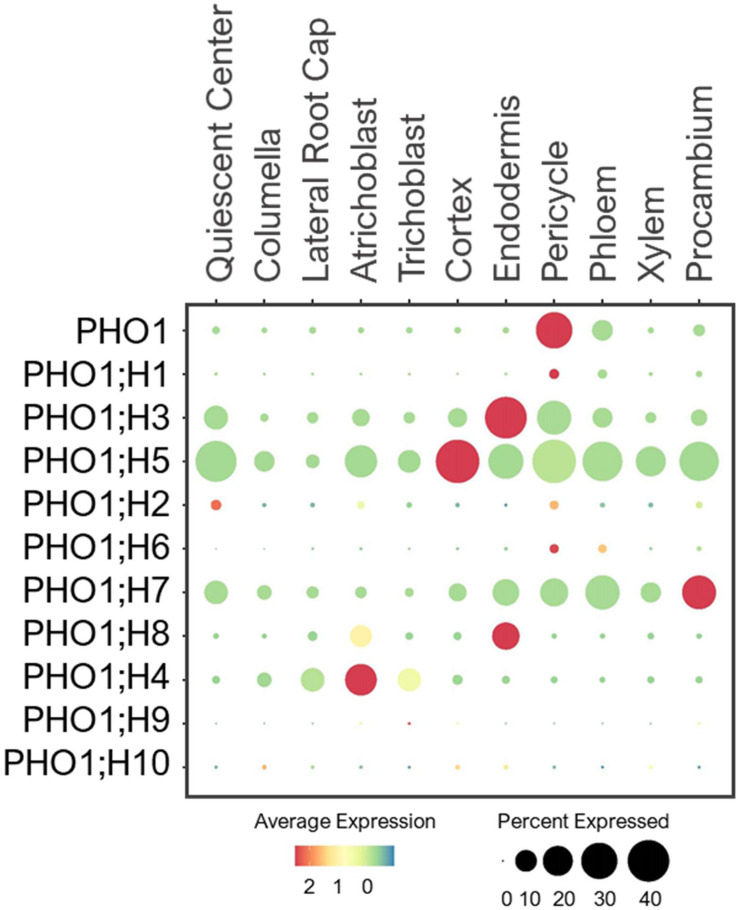
Expression of the PHO1 transporters in Arabidopsis root single cells. In dot plot, the circle size represents the proportion (%) of cells expressing a given gene, and the color of scale bar represents the mean expression (natural log +1 pseudocount).

Plants transfer Pi from root-to-shoot via xylem loading. This process requires Pi efflux from root pericycle to xylem, which is mediated by the Golgi-localized Pi exporter, PHO1 (Poirier et al., 1991; Hamburger et al., 2002; Stefanovic et al., 2011; Arpat et al., 2012). It contains 10 additional homologs in the Arabidopsis genome, referred to as PHO1;H1–H10 (Wang et al., 2004). Studies have shown their tissue- and organ-specific expressions (Hamburger et al., 2002; Wang et al., 2004; Stefanovic et al., 2007; Khan et al., 2014), but their cell-type-specific expression in the root system remains unknown. PHO1;H1 is closely related to PHO1, and shares a redundant function in xylem loading (Stefanovic et al., 2007). Consistent with their functions, single cell profiling revealed the overlapping expression of PHO1 and PHO1;H1 in pericycle (Figure 8). However, PHO1;H1 was expressed at a low level under the normal conditions because its function is more prominent in response to low-Pi (Stefanovic et al., 2007).

PHO1;H3 shared a similar expression pattern and subcellular localization to PHO1, but it had the opposite function in long-distance transport (Hamburger et al., 2002; Khan et al., 2014). It was shown to restrict root-to-shoot Pi transport under zinc deficiency (Khan et al., 2014). In root single cell profile, PHO1;H3 was largely expressed in endodermis and pericycle (Figure 8). This implies PHO1;H3 may retrieve Pi from the xylem back to pericycle and endodermis. PHO1;H2, PHO1;H5 and PHO1;H6 are close homologs of PHO1;H3 (Wang et al., 2004), and shared overlapping expressions in pericycle (Figure 8). However, PHO1;H2 and PHO1;H6 were expressed at low level and may require specific environmental conditions to induce their expression and function in long-distance transport. In contrast, PHO1;H5 was broadly expressed in all cell-types except root cap, suggesting its potential role in radial Pi distribution from root epidermis to stellar cells or vice versa. PHO1;H4, PHO1;H7, and PHO1;H8 are closely related (Wang et al., 2004), but they showed distinct expression patterns. For example, PHO1;H4 was largely expressed in epidermis and lateral root cap (Figure 8), and may mediate root-soil Pi exchange. PHO1;H8 was highly expressed in endodermis, and likely transport Pi to pericycle for xylem loading. PHO1;H7 was more broadly expressed in endodermis and stellar cells, suggesting its role in xylem and/or phloem loading. However, these are putative functions based on their expression patterns, and need to be validated experimentally. Whether all these PHO1 homologs localize to Golgi, and how Golgi might engage in long-distance transport remain unknown.

#### Potential Network of Phosphate Transporters in Roots

**FIGURE 9:**
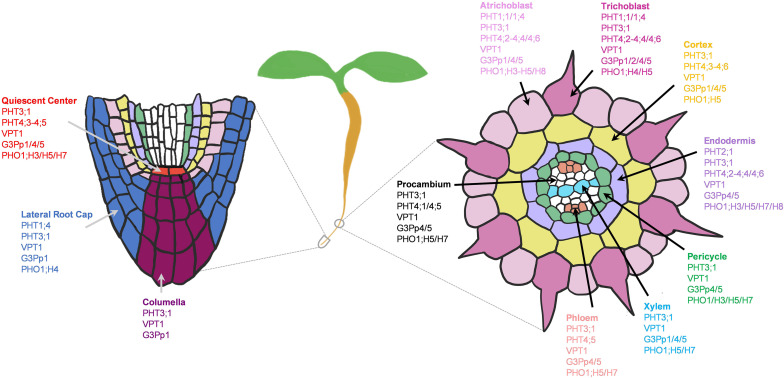
Potential network of phosphate (Pi) transporters in the root system of 5–7 days old Arabidopsis seedlings grown under the normal growth conditions. Expressed Pi transporters involved in longitudinal transport (left) at the meristematic zone and radial transport (right) at the elongation/maturation zones were shown. Pi transporter genes not expressed under the normal growth conditions were excluded.

Pi transporters are found at the PM and membranes of different subcellular compartments, and most of their tissue expressions have been elucidated (Wang et al., 2004; Ramaiah et al., 2011; Gu et al., 2016; Luan et al., 2017; Xu et al., 2019). However, many of their cell-type specific expressions, physiology and Pi transport network in the root system are not well understood. By examining the expression patterns of Pi transporters, we generated a potential network of Pi transporters involved in longitudinal and radial Pi transport in the root system of young Arabidopsis seedlings under the normal growth conditions (Figure 9). In general, PHT1s (esp. PHT1;1/1;4) mainly absorbed Pi from the root tip and epidermis, while other subcellular-localized PHT, G3Pp, and PHO1;H transporters might distribute Pi further to the adjacent cells. Many of these subcellular Pi transporters that are expressed under the normal conditions showed widespread expression to distribute Pi throughout the root system to support housekeeping functions. These include mitochondrial PHT3;1, vacuolar PHT5;1, most plastid/Golgi PHT4s, putative plastid G3Pp4/5, and putative Golgi PHO1;H5.

Several Pi transporters showed cell-type specific expressions, including G3Pp2 and PHO1;H4 in epidermis, PHT2;1 in endodermis, and PHO1 in pericycle. Among these, G3Pp2 is shown to be involved in vacuolar Pi remobilization (Xu et al., 2019), PHT2;1 in Pi transport into plastids (Daram et al., 1999; Versaw and Harrison, 2002; Rausch et al., 2004), and PHO1 in root-to-shoot Pi translocation (Poirier et al., 1991; Hamburger et al., 2002). Except for PHO1, their contributions in specific root cell-types remain obscure, and may be subjected to modifications upon the changing environmental stresses and developmental stages. We additionally found several PHO1 homologs especially PHO1;H5/H7 that are most likely involved in long-distance transport during plant development (Figure 9). These are potential candidates for both xylem and/or phloem loading or unloading, which need to be confirmed genetically and physiologically.

To understand how these Pi transporters may contribute to the low-Pi adaptation, we also profiled their expression patterns from scRNA-seq analyzed under low-Pi (Wendrich et al., 2020). PHT1;1/1;4, PHT2;1, VPT1, G3Pp2, PHO1, and PHO1;H3/H5 were differentially expressed in response to low-Pi (Supplementary Figure 1), with similar expression patterns to our analysis. These Pi transporters may contribute to low-Pi adaptation, probably by enhancing Pi uptake, fine-tuning Pi fluxes from subcellular compartments, and redistributing Pi between shoots and roots. Therefore, these Pi transporters are worth further investigations especially PHT2;1, G3Pp2, and PHO1;H5, whose roles in the root system have not been studied yet. In addition, we need to identify novel Pi transporters that transfer Pi across the PM of different root cell-types besides PHT1s.

### Potassium Transport

K^+^ is the most abundant cation in plants, required for the membrane potential maintenance, protein synthesis, enzyme activation, stomatal movement and photosynthesis (Wang et al., 2013; Luan et al., 2017; Wang and Wu, 2017; Hasanuzzaman et al., 2018). Plants need 100–200 mM of cytosolic K^+^ to sustain cell metabolism (Walker et al., 1996; Tester and Leigh, 2001; Sharma et al., 2013; Luan et al., 2017). However, K^+^ is often limited in the natural environment (Maathuis, 2009; White, 2013), leading to heavy fertilizer application that raises sustainability issues. To combat the large fluctuations in the soil K^+^, plants have evolved elaborate transport systems to maximize K^+^ absorption and distribution. These include *Shaker*-type and TPK (Tandem Pore K^+^) channels, KUP/HAK/KT (K^+^ Uptake/High-Affinity K^+^ Transporter) transporters, and four families of Cation/Proton Antiporters (CPA), including CHX (Cation/H^+^ Exchangers), KEA (K^+^ Efflux Antiporters) and NHX (Na^+^/H^+^ Exchangers) (Mäser et al., 2001; Gomez-Porras et al., 2012; Wang and Wu, 2013; Santa-María et al., 2018; Lhamo et al., 2021). Decades of research have elucidated the importance of these K^+^ channels and transporters in different plant tissues and organs. However, how these diverse K^+^ channels and transporters coordinate in the root system to translocate K^+^ to aerial tissues is unclear. We utilize the single cell profiles to provide a potential network of K^+^ channels and transporters that transport K^+^ from the root epidermis to the xylem vessels using young Arabidopsis seedlings grown under the normal conditions.

#### *Shaker*-Type Channels

**FIGURE 10:**
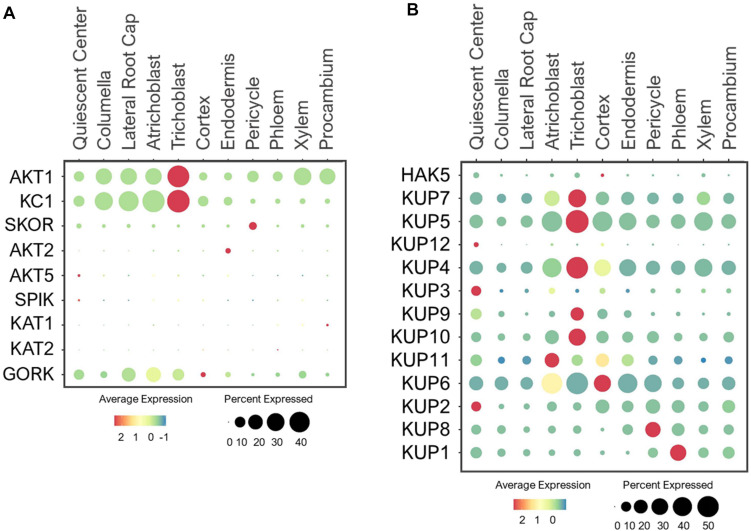
Expression of the **(A)** *Shaker-*type channels **(B)** KUP transporters in Arabidopsis root single cells. In dot plots, the circle size represents the proportion (%) of cells expressing a given gene, and the color of scale bar represents the mean expression (natural log +1 pseudocount).

The Arabidopsis *Shaker*-type family is a well-studied group of voltage-gated K^+^ channels, with nine members that are classified into three categories based on their voltage dependence (Pilot et al., 2003; Lebaudy et al., 2007; Wang and Wu, 2013). These include K^+^ inward-rectifying channels such as AKT1/5, KAT1/2, and SPIK (Hirsch et al., 1998; Pilot et al., 2001; Mouline et al., 2002), and weak-rectifying channel, AKT2 (Lacombe et al., 2000; Michard et al., 2005) that are activated by membrane hyperpolarization; and outward-rectifying channels such as SKOR and GORK that are activated by membrane depolarization (Gaymard et al., 1998; Ache et al., 2000). These *Shaker*-type channels are mainly localized to PM and participate in different physiological processes (Wang and Wu, 2013; Véry et al., 2014; Luan et al., 2017; Lhamo et al., 2021). For example, AKT1 channel is known to mediate K^+^ uptake from the soil (Lagarde et al., 1996; Hirsch et al., 1998; Gierth et al., 2005), and its activity is fine-tuned by KC1 channel via heteromerization (Reintanz et al., 2002; Duby et al., 2008; Geiger et al., 2009a). As such, these two K^+^ channels shared overlapping expression patterns in the root tip and epidermis (Figure 10A), where changes in K^+^ level can be sensed to initiate or prevent K^+^ uptake. AKT1 was additionally expressed in large portions of xylem and procambium, suggesting its additional role in K^+^ translocation. This is in line with a previous study reporting AKT1 involvement in K^+^ retrieval from the xylem sap under the sufficient-K^+^ condition (Nieves-Cordones et al., 2019).

For shoot translocation, K^+^ efflux channel, SKOR mediates K^+^ release from pericycle to xylem (Gaymard et al., 1998; Liu et al., 2006). As expected, SKOR was expressed specifically in pericycle (Figure 10A). However, its low expression level implies the existence of other transporters that may also function in K^+^ root-to-shoot translocation under the normal conditions since the K^+^ concentrations in shoots were previously reported to be twice the roots in different plant species using the K^+^-selective microelectrodes (Tester and Leigh, 2001). Once K^+^ is present in leaves, it can be recirculated back to roots by AKT2 via phloem loading (Marten et al., 1999; Lacombe et al., 2000). However, AKT2 was lowly detected in root single cells (Figure 10A), suggesting a minimal requirement for K^+^ recycling from shoot-to-root under the normal conditions. This recycling process may be activated during low-K^+^ stress to support root metabolic processes.

Several *Shaker*-type channels were shown to be specifically expressed and function in green tissues and floral organs. For example, AKT5 and SPIK displayed flower-specific expression, where SPIK channel was reported to conduct K^+^ influx into pollen for pollen tube growth (Lacombe et al., 2000; Mouline et al., 2002; Zhao et al., 2013). KAT1/2 are expressed in leaves and flowers, where they mediate K^+^ influx to control stomatal opening (Nakamura et al., 1995; Kwak et al., 2001; Pilot et al., 2001). Since these four *Shaker*-type channels are specifically expressed in aerial tissues, their expressions in root single cells were barely detected (Figure 10A). GORK is also expressed in leaf stomata and mediates K^+^ efflux to induce stomatal closure (Ache et al., 2000; Hosy et al., 2003). Studies have also revealed GORK expression in root epidermis, where it mediates K^+^ efflux from root cells to maintain osmotic balance (Ivashikina et al., 2001; Osakabe et al., 2013; Demidchik, 2014). We also found GORK to be expressed in large portions of epidermal, LRC and QC cells (Figure 10A), suggesting GORK may export K^+^ radially and longitudinally from the root surfaces. Because AKT1 and GORK expressions overlap, their coordination at the root surface might balance K^+^ influx and efflux, which may prevent epidermal and LRC cells from bursting under the nutrient-rich conditions.

#### KUP Transporters

The KUP family encodes 13 KUP/HAK/KT members in Arabidopsis that show high homology to the bacterial KUP and fungal TRK transporters (Schleyer and Bakker, 1993; Banuelos et al., 1995). Similar to *Shaker*-type channels, these KUP transporters also have diverse roles in transporting K^+^ throughout different cells and tissues. In addition, KUPs are localized to the PM and different subcellular compartments, and some are capable of transporting hormones (Li et al., 2018; Lhamo et al., 2021). Among these, the PM-localized HAK5 and KUP7 mediate K^+^ uptake in response to low-K^+^ stress, with an additional role of KUP7 in K^+^ translocation to shoots (Gierth et al., 2005; Qi et al., 2008; Pyo et al., 2010; Han et al., 2016). Since HAK5 functions specifically under low-K^+^ (10–100 μM) stress (Rubio et al., 2008; Nieves-Cordones et al., 2014), its expression was hardly detectable in young roots under the normal conditions (Figure 10B). However, KUP7 exhibited ubiquitous expression, suggesting its ability to transport K^+^ from epidermal to stellar cells for shoot translocation during the normal growth. Although an earlier study indicated probable role of HAK5 in K^+^ translocation under the sufficient-K^+^ condition (Nieves-Cordones et al., 2019), our single cell profiles indicated more probable role of KUP7 than HAK5 in this process during early plant development. HAK5 may be involved in K^+^ translocation during the later stages of development or specially under low-K^+^ stress, requiring more detailed analysis. Like KUP7, its close homolog, KUP5, was also ubiquitously expressed (Figure 10B), suggesting these two KUPs may functionally overlap with AKT1 in K^+^ uptake and SKOR in K^+^ translocation during normal growth conditions.

In addition to K^+^ transport, several KUPs function in root development. For instance, KUP4 (TRH1) located in the endomembrane promotes root hair growth and gravitropic responses by modulating auxin transport in the root apex (Rigas et al., 2001, 2013; Vicente-Agullo et al., 2004). The single cell profile indicated that KUP4 was ubiquitously expressed in young roots, suggesting it’s important role in root development (Figure 10B). The high expression of KUP4 in trichoblast and cortex may suggest its involvement in generating auxin maxima needed for root hair elongation (Jones et al., 2009; Vissenberg et al., 2020). This is supported by genetic analyses of the KUP4 null mutants that display no root hair growth, and reduced auxin accumulation in epidermal and cortical cells compared to the wild type upon exogenous auxin treatment (Rigas et al., 2013). In addition, the expression of KUP4 in stellar cells suggests it may be involved in both acropetal and basipetal auxin distribution (Sustr et al., 2019). Its close homolog, KUP3, was predominantly expressed in QC (Figure 10B), therefore, may fine-tune K^+^ and/or hormone distribution to support meristematic activity as observed for KUP9. Recently, KUP9 is shown to mediate auxin and K^+^ export from ER (Endoplasmic reticulum) to cytosol in response to low-K^+^, which is essential to maintain meristematic activity and primary root growth (Zhang et al., 2020). As expected, KUP9 was expressed in QC, but also in trichoblast and cortex (Figure 10B). This suggests KUP9 may also contribute to polar auxin transport and K^+^ distribution during plant growth. The closely related KUP10 and KUP11 displayed broad expressions, however, their single mutants did not show low-K^+^ sensitive phenotype observed for *kup9* mutants (Zhang et al., 2020). Generation of higher-order mutants may dissect functional redundancies between these close homologs, with KUP9 as the dominant player.

KUP2/6/8 are K^+^ efflux transporters that are suggested to function redundantly in exporting K^+^ from stellar cells to epidermis (Osakabe et al., 2013). Consistent with this study, our single cell profiling revealed overlapping expressions of these KUPs in stellar cells, with the broader expression of KUP6 in other cell types (Figure 10B). The coordination among KUP2/6/8 might allow efficient K^+^ transport from stellar cells to root epidermis, where KUP6 along with GORK likely extrude K^+^ from roots back to the soil. It remains a question whether young plants use such a mechanism to remove K^+^ buildup under the sufficient-nutrient availability for osmotic balance. In addition, analysis of *kup2/6/8* triple mutants revealed the importance of these KUPs in hormone responses that affect cell expansion and lateral root development (Osakabe et al., 2013), however, the mechanism is unclear. Similar to KUP4/9, we suspect that KUP2/6/8 may be capable of transporting hormones in addition to K^+^. KUP1, the founding member of the Arabidopsis KUP family, was previously shown to mediate both high- and low-affinity K^+^ transport (Fu and Luan, 1998; Kim et al., 1998). However, its tissue expression and physiological role have not been examined. Our study indicated KUP1 expression in stellar cells, especially the phloem in young seedlings (Figure 10B). This implicated the possible role of KUP1 in phloem loading, and may work in parallel or substitute AKT2 function under the normal growth conditions. Genetic and physiological analyses are needed to confirm its putative function.

#### CHX Antiporters

**FIGURE 11:**
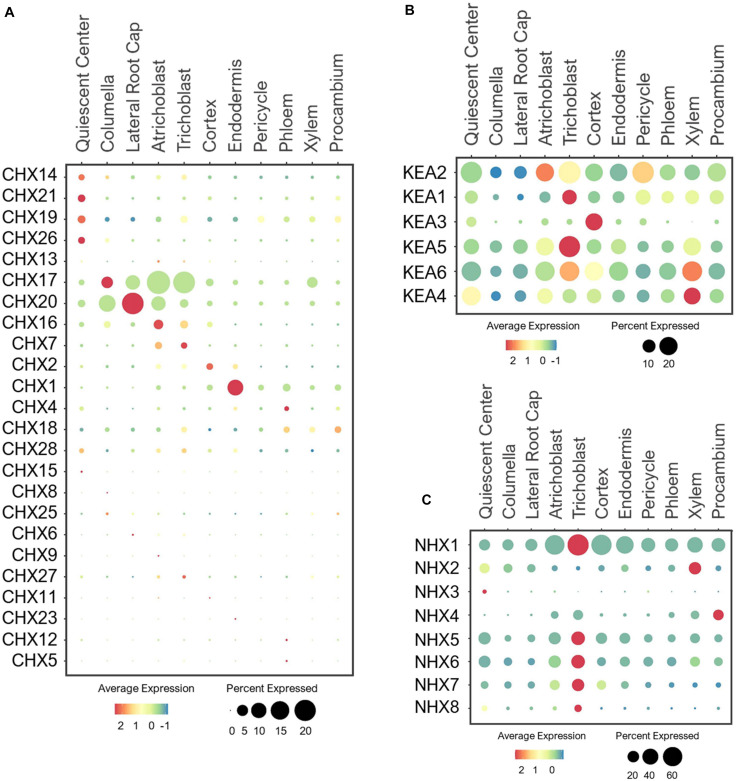
Expression of the **(A)** CHX, **(B)** KEA and **(C)** NHX antiporters in Arabidopsis root single cells. In dot plots, the circle size represents the proportion (%) of cells expressing a given gene, and the color of scale bar represents the mean expression (natural log +1 pseudocount).

CHX is the largest family belonging to CPA superfamily, which in general plays important role in regulating pH and ion homeostasis of the subcellular organelles that affect protein processing, vesicular cargo composition, vesicle movement and protein trafficking (Bassil et al., 2012; Sze and Chanroj, 2018). The CHX family consists of 28 members in Arabidopsis, which are localized to the PM and endosomes where they perform K^+^/H^+^ exchange (Chanroj et al., 2011; Sze and Chanroj, 2018). The physiological functions of many of these CHXs remain uncharacterized. Earlier studies showed that a large number of CHXs transporters are expressed in pollen, among which, some are functionally characterized to be involved in male fertility, pollen tube guidance, pollen wall construction and seed development (Sze et al., 2004; Lu et al., 2011; Chanroj et al., 2013; Sze and Chanroj, 2018). Because our single cell profiling is focused on roots of developing seedlings, the expression of many of these CHXs were detected at low levels (Figure 11A).

However, several CHX members are known to be expressed and function in the root system. These include the PM-localized CHX13 and CHX17 that mediate high-affinity K^+^ uptake under low-K^+^ (Cellier et al., 2004; Zhao et al., 2008). While expression of CHX13 was not detected in the root under the normal conditions, CHX17 was highly expressed in the epidermis and root cap (Figure 11A). This implies CHX17, but not CHX13, may mediate K^+^ uptake in developing seedlings under the normal conditions. CHX13 is probably more responsive to low-K^+^ stress, similar to HAK5. CHX20 was previously shown to mediate K^+^/H^+^ exchanges in ER to control the stomatal movement (Padmanaban et al., 2007), but it’s role in roots have not been elucidated. We found CHX20 to be highly expressed in the root cap and atrichoblast, and may control K^+^ fluxes in ER of these cells, likely to regulate protein processing and trafficking as reported (Chanroj et al., 2011). CHX7 and CHX16 were expressed at low levels in epidermis (Figure 11A), and may also participate in K^+^ uptake or distribution depending on their membrane localizations. Between the two, CHX16 was shown to be located at the PM and ER, and implicated to function in pH homeostasis (Chanroj et al., 2011, 2013). In maintaining pH, CHX16 at the PM of root epidermis is likely to initiate K^+^ acquisition in exchange.

Several CHXs have been reported to function in response to osmotic stresses. For example, the PM-localized CHX14 is suggested to mediate K^+^ efflux from vascular stellar cells under excess-K^+^ condition (Zhao et al., 2015). On the other hand, the PM-localized CHX21 in root endodermis is implicated in loading Na^+^ to stelar cells under salinity stress for sequestration into leaf vacuoles (Hall et al., 2006). These stress-responsive transporters, CHX14/21, were lowly detected in QC under the normal conditions, similar to CHX19/26 (Figure 11A). Based on their known functions, the QC-specific expression might be an artifact. It is more likely that CHX14/21 expression and function may be more pronounced in stellar cells under osmotic stresses as reported (Hall et al., 2006; Zhao et al., 2015). CHX1 was the only member in the CHX family to be highly expressed in the endodermis (Figure 11A), raising the interesting possibility for this CHX to play a role in long-distance transport.

#### KEA Antiporters

The KEA family is also part of the CPA superfamily, with six members that share high homology to the bacterial K^+^/H^+^ antiporters KefB and KefC (Mäser et al., 2001; Chanroj et al., 2012; Han et al., 2015; Sze and Chanroj, 2018). Previous studies reported their tissue-specific expressions (Han et al., 2015; Zhu et al., 2018), however, their expressions at a cellular level are unclear. At the subcellular level, the closely related KEA1/2 are located at the inner envelope membrane and KEA3 at the thylakoid membrane of chloroplasts (Kunz et al., 2014). These KEAs participate in the K^+^/H^+^ exchange within and across chloroplasts to maintain pH and osmotic balance for chloroplast development and photosynthesis (Mäser et al., 2001; Kunz et al., 2014). Interestingly, we detected a broad expression of KEA1/2 in multiple cell-types in developing roots under the normal growth conditions (Figure 11B). This suggests the possible roles of KEA1/2 in K^+^ and pH homeostasis of plastids in different cell-types of roots in addition to shoots. In contrast, the more distant member, KEA3, was exclusively expressed in the cortex, and may mediate K^+^/H^+^ exchange specifically in the cortical plastids, which require further analysis to understand its functional significance in this specific cell-type.

The closely related KEA4/5/6 mediate K^+^/H^+^ exchange across trans-Golgi networks for proper vesicle trafficking that allow better adaptation to various osmotic stresses (Zhu et al., 2018; Wang et al., 2019). These KEAs shared overlapping and ubiquitous expressions in young roots under the normal growth conditions (Figure 11B), emphasizing their importance in pH/ionic homeostasis to support the endomembrane function in all root cell-types. Although broadly expressed, most KEAs displayed the highest expression in root epidermis, mainly trichoblast. It would be interesting to examine if these KEAs have any specific roles in root hair development. KEAs were also widely expressed in stellar cells, with the high expression of KEA2 detected in pericycle and KEA4/5/6 in xylem. This is in agreement with the previous findings (Han et al., 2015), suggesting these KEAs may further contribute to K^+^ distribution across stellar cells for shoot translocation. Currently, the physiological function of KEA members in the root system, and their possible involvement in K^+^ partitioning between roots and shoots have not been elucidated. In addition, how these KEAs may allow adaptation to low-K^+^ and excess-K^+^ stresses at the tissue- and cell-specific levels need to be further evaluated since their higher-order mutants (e.g., *kea4/5/6)* were highly sensitive to these K^+^ stresses (Zhu et al., 2018; Wang et al., 2019).

#### NHX Antiporters

The NHX family is another group of the CPA superfamily. In Arabidopsis, there are eight members, originally described as Na^+^/H^+^ exchangers (thus NHX) (Blumwald and Poole, 1985). Later studies indicated that different NHX members can transport Na^+^, K^+^, or both in exchange of H^+^ (Venema et al., 2002; Liu et al., 2010; Bassil et al., 2011b; Barragán et al., 2012). These transporters are found at the PM and different subcellular compartments (Bassil et al., 2012). NHX1/2 are located at the tonoplast to mediate K^+^ sequestration into the vacuoles (Shi and Zhu, 2002; Bassil et al., 2011b; Barragán et al., 2012). In aerial parts, they regulate stomatal movement and flower development (Bassil et al., 2011b; Barragán et al., 2012). We found that NHX1 was ubiquitously expressed in various cell-types of roots, whereas NHX2 showed narrower expression in xylem and QC (Figure 11C). This is consistent with the previous reports of tissue-specific expressions (Shi and Zhu, 2002; Barragán et al., 2012). The expression dominance of NHX1 compared to NHX2 coincides with its functional dominance in maintaining turgor for cell expansion to support overall growth and development (Bassil et al., 2011b). Because NHX2 was highly expressed in xylem, it may be involved in maintaining high K^+^ pools necessary for shoot translocation when plants are challenged by K^+^ limitations. Their roles in the root system need to be further evaluated because the earlier studies mainly focus on aerial tissues, where NHX1/2 were also expressed. The K^+^-selective microelectrode analysis revealed the maintenance of constant cytosolic K^+^ concentration between different root cell-types under the sufficient-K^+^ condition, and suggested the involvement of vacuolar transporters in this process (Walker et al., 1996; Tester and Leigh, 2001). The vacuolar NHXs are the prime candidates to sequester the accumulating K^+^ under the nutrient sufficiency.

NHX3 and NHX4 are also localized to tonoplast, with the NHX3 expression detected in pollen grains and siliques, and NHX4 in roots and shoots (Li et al., 2009; Liu et al., 2010; Bassil et al., 2012). Consistently, NHX3 was barely detected in roots (Figure 11C), suggesting it may have developed a specific function in reproductive organs. In contrast, the NHX4 was highly expressed in procambium, where it may provide the K^+^ supply needed for procambium differentiation into mature xylem and phloem. Similar to NHX2, NHX4 may support a long-distance K^+^ transport to shoots. Its contribution to K^+^ nutrition in the root system needs to be further explored. One important reason is because a previous study reported the highest K^+^ concentration in vacuoles of xylem compared to all other cell types, followed by pericycle, endodermis and cortex by using the cryo-scanning microscopy with energy dispersive X-ray microanalysis (McCully, 1994). NHX1/2/4 may be the key players in maintaining the high K^+^ level in xylem.

NHX5 and NHX6 are closely related transporters with high homology to the yeast NHX1 and the human NHE6 and NHE7 (Khan et al., 2018). These NHXs maintain pH and K^+^/Na^+^ homeostasis in endosomes to mediate cell expansion, proliferation and vesicle trafficking by influencing auxin transport and distribution (Bassil et al., 2011a; Dragwidge et al., 2018, 2019). NHX5/6 shared broad expression in the root single cell profiles (Figure 11C), supporting their essential and redundant functions in the housekeeping processes. The PM-localized NHX7 (SOS1) and NHX8 are the most divergent members of the NHX family, which closely resemble the prokaryotic NhaP antiporter (Bassil et al., 2012). These NHXs maintain cellular ion homeostasis by extruding toxic Na^+^ and Li^+^ out of the root cells (Shi et al., 2002; An et al., 2007). Consistent with their functions, NHX7/8 were highly expressed in epidermis, especially trichoblast, to remove the toxic cations out of the cell. In return, K^+^ may serve as a counter ion of Na^+^ to be absorbed by NHX7 at the root surface (Wu et al., 1996).

#### TPK Channels

**FIGURE 12:**
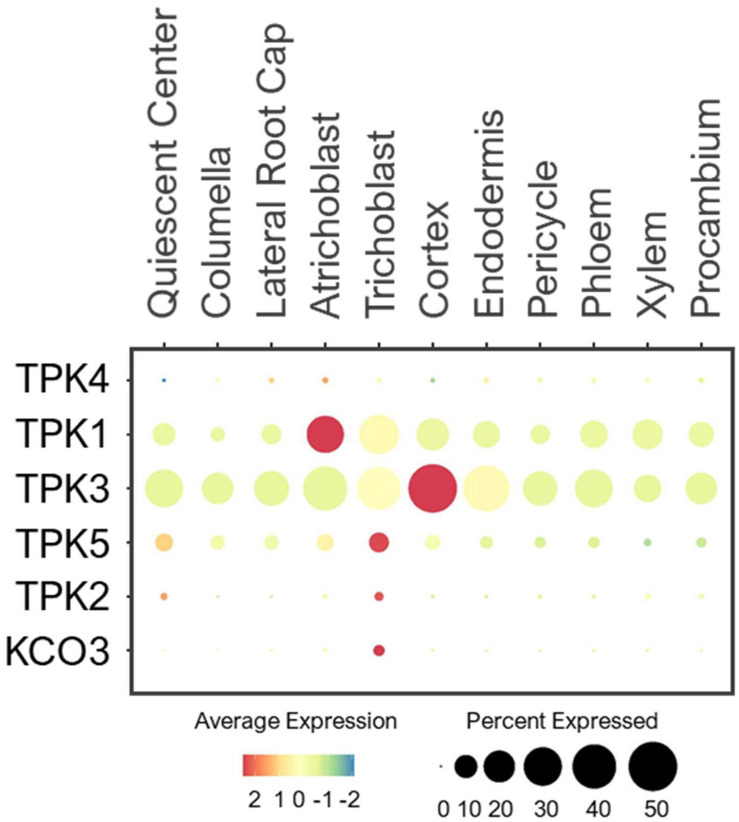
Expression of the TPK channels in Arabidopsis root single cells. In dot plot, the circle size represents the proportion (%) of cells expressing a given gene, and the color of scale bar represents the mean expression (natural log +1 pseudocount).

The TPK family is composed of five Tandem Pore K^+^ channels (TPKs) and one K_ir_-like K^+^ channel. All of them are localized to the tonoplast except TPK4, which is found at the PM (Voelker et al., 2006, 2010; Lhamo et al., 2021). Electrophysiological studies in heterologous systems revealed TPK1/4 to be voltage-independent, K^+^ selective channels, and their channel activities are regulated by calcium and pH (Becker et al., 2004; Bihler et al., 2005; Gobert et al., 2007; Tang et al., 2020). TPK4 is expressed predominantly in pollen, where it conducts K^+^ across the PM of pollen tubes (Becker et al., 2004). As reported, the expression of TPK4 was barely detected in root cells of young seedlings under the normal conditions (Figure 12). On the other hand, the tonoplast-localized TPK1 is ubiquitously expressed, and plays important role in low-K^+^ adaptation by releasing K^+^ from vacuoles (Czempinski et al., 2002; Bihler et al., 2005; Gobert et al., 2007; Tang et al., 2020). The physiological characterizations of other tonoplast-localized TPKs remain unelucidated. In root single cells, TPK1/3/5 were widely expressed in all cell-types although TPK5 was expressed at a lower level (Figure 12). This suggests these TPKs may share similar housekeeping functions in vacuolar K^+^ remobilization throughout the root system. While their functions are likely to dominate under low-K^+^ stress, TPK1/3/5 may modulate the vacuolar K^+^ fluxes in coordination with NHX1/2 to maintain the constant cytosolic K^+^ concentration under the normal growth conditions, similar to AKT1 and GORK at the PM. The tonoplast-localized TPK2 and KCO3 were lowly detected in trichoblast under the normal conditions. Their expressions may amplify in multiple root cell-types under low-K^+^, to optimize K^+^ remobilization along with TPK1/3/5. However, the presence of all vacuolar TPKs at the root epidermis might explain the previous findings that cytosolic K^+^ concentration declines faster in epidermal cells than cortical cells upon continual low-K^+^ treatment (Walker et al., 1996; Tester and Leigh, 2001).

#### Potential Network of Potassium Channels and Transporters in Roots

**FIGURE 13:**
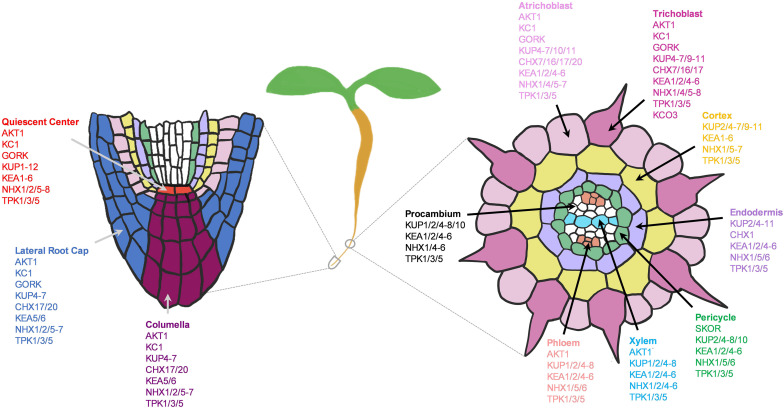
Potential network of potassium (K^+^) channels and transporters in the root system of 5–7 days old Arabidopsis seedlings grown under the normal growth conditions. Expressed K^+^ transporters involved in longitudinal transport (left) at the meristematic zone and radial transport (right) at the elongation/maturation zones were shown. K^+^ transporter genes not expressed under the normal growth conditions were excluded.

Single cell profiling has allowed us to generate a working model of how diverse K^+^ channels and transporters might coordinate to transport K^+^ across different root cell-types of developing seedlings under the normal growth conditions (Figure 13). However, these are purely based on transcriptional profiles, which is subjected to change upon different developmental stages and K^+^ status. In general, K^+^ uptake and long-distance transport are largely mediated by *Shaker*-type channels, KUP and CHX transporters. The subcellular K^+^ distribution is controlled by TPK channels, KUP, KEA, NHX, and CHX transporters. Although many of their membrane localizations were elucidated, the functional studies of these K^+^ channels and transporters in the root system await further investigations. At the root surfaces, AKT1, HAK5, KUP7, and CHX13/17 are known to participate in K^+^ uptake especially in response to low-K^+^ stress (Hirsch et al., 1998; Cellier et al., 2004; Gierth et al., 2005; Qi et al., 2008; Zhao et al., 2008; Han et al., 2016), whereas KC1 channel inhibited AKT1 activity (Reintanz et al., 2002; Duby et al., 2008; Geiger et al., 2009a). Our expression analysis indicated AKT1, KUP7, and CHX17 to also mediate K^+^ acquisition under the normal growth conditions in young roots. We additionally found a number of KUP, CHX, KEA, NHX transporters, and TPK channels expressed at the root tip and epidermis that might be involved in K^+^ influx and efflux into and out of the root cells and subcellular compartments (Figure 13). Among these, several showed cell-type specific expressions at low level, including KUP2/3 in QC and TPK2/KCO3 in trichoblast. Several KUP, KEA, NHX, and TPK members were expressed in cortex and endodermis to distribute K^+^ within and toward stellar cells (Figure 13). KEA3 was predominantly expressed in the cortex and CHX1 in the endodermis, suggesting their unique functions in these cell-types, which might be worth further investigations.

In stellar cells, SKOR and KUP7 were known to mediate xylem loading (Gaymard et al., 1998; Liu et al., 2006; Han et al., 2016) AKT2 in phloem loading (Marten et al., 1999; Lacombe et al., 2000), and AKT1 in xylem retrieval (Nieves-Cordones et al., 2019). Surprisingly, SKOR and AKT2 channels were lowly expressed in developing roots under the normal conditions; therefore, we anticipated the involvement of additional nutrient transporters in these processes. For instance, NPF7.3 (NRT1.5) in pericycle is shown to mediate K^+^ loading to xylem under low-K^+^ and the normal conditions, although its function is more pronounced under low-nutrient (K^+^ or NO_3_^–^) stresses (Drechsler et al., 2015; Li et al., 2017b). In addition to this, we found number of KUPs, KEAs, NHXs, and TPKs that might contribute to long-distance K^+^ transport (Figure 13). Among these, NHX2/4 might be more specifically involved in xylem loading and KUP1 in phloem loading. KUP2/6/8 and GORK might coordinate with each other to extrude K^+^ from stellar cells to the soil (Osakabe et al., 2013).

Overall, many KUPs were widely expressed, indicating their essential role in K^+^ transport in developing roots under the sufficient-K^+^ supply, and worth further studies to dissect the function of uncharacterized KUPs. Because there are large number of KUPs, functional redundancies among the KUP transporters may restrict further studies, which could be potentially resolved by generating higher-order mutants. Our analysis provides KUP genes that share overlapping expressions that may be used to generate higher-order mutants. KEAs are essential for K^+^/H^+^ balance in plastids and endomembranes, but their significance in K^+^ transport and distribution in the root system remain unelucidated despite their broad expressions. On the other hand, the large portions of *Shaker*-type channels and CHX transporters were not much expressed in young roots, suggesting their roles in other tissues and organs or in response to specific biotic and abiotic stresses. Several NHX members were ubiquitously expressed for housekeeping functions in tonoplast and endosomes. TPK1/3/5 were broadly expressed to remobilize K^+^ from vacuoles, and may function concurrently with the vacuolar influx transporter, NHX1 in maintaining cellular K^+^ homoeostasis during plant development. However, TPK3/5 are not physiologically characterized yet, and more studies of NHX members in roots are needed.

While we focus on the major families of K^+^ channels and transporters, there exist other voltage-independent, non-selective cation channels such as CNGCs (cyclic nucleotide gated channels) that are capable of transporting K^+^ in different heterologous systems, which include CNGC1/2/3/4/10/17 (Köhler et al., 1999; Leng et al., 1999; Balagué et al., 2003; Hua et al., 2003; Li et al., 2005; Gobert et al., 2006; Ladwig et al., 2015). This shows the complexity of K^+^ transport in plants, in which various channels and transporters are able to control K^+^ fluxes during different developmental stages and in response to various environmental stresses. Nevertheless, they share a similar goal of providing K^+^ supply needed to support growth, which involves the coordination among different nutrient transporters and channels to maintain cellular ion homeostasis.

## Conclusion

In summary, single cell profiling has allowed us to predict the functions of many unknown NPK channels and transporters and generate their potential networks in the root system of young Arabidopsis seedlings. However, further molecular, electrophysiological and genetic studies are required to confirm their functions in the specific root cell-types, and determine functional redundancies among the closely-related transporters. In addition, generation of root atlas using a large population of cells under different developmental and low-nutrient stresses may unravel the putative channel and transporter networks that are crucial for plant development and low-nutrient adaptation. Moreover, the spatial profiling of cytosolic and subcellular nutrient concentrations in root single cells using technologies such as microelectrodes (Zhen et al., 1991; Radcliffe et al., 2005), nuclear magnetic resonance (NMR) (Lee and Ratcliffe, 1993; Radcliffe et al., 2005; Gout et al., 2011; Liu et al., 2016) and fluorescence resonance energy transfer (FRET)-based systems (Looger et al., 2005; Mukherjee et al., 2015; Sahu et al., 2020) are much needed to dissect the complexity of nutrient transport within and between different root cell-types.

## Materials and Methods

### Plant Growth Conditions for Low-Nutrient Stresses

Arabidopsis seeds were sterilized in 10% bleach, rinsed with distilled water four times, and plated directly on 1/6 strength MS (Murashige and Skoog) with (NPK) and without N, P, or K (0 mM) nutrients, supplemented with 1% sucrose and 1% phytoblend (Caisson labs), pH 5.7. The MS media with and without N or P were ordered from Caisson labs (North Logan, Utah). Low-K medium (1/6 MS) were prepared in-house using 0.5 mM Ca(NO_3_)_2_, 0.25 mM MgSO_4_, 0.21 mM NH_4_H_2_PO_4_, and 1/6 micronutrients (Caisson labs). Plated seeds were stratified at 4°C for 2 days and transferred to the growth chamber in vertical position for 5 days, with a 12-h light/12-h dark cycle (100 μmol m^–2^ s^–1^). Two sets of plates were prepared for each condition, one with plants clustered together, and the other with seedlings separated with spaces in-between for root hair phenotype, and the most representative one is presented.

### Single-Cell Root Altas Data Source and Information

The scRNA-seq data and Seurat objects for Arabidopsis root altas were obtained from Shahan et al. (2020) (GEO: GSE152766). Briefly, the primary roots of 5–7 days old Arabidopsis seedlings under normal growth conditions were used for root protoplast extractions. Shahan et al. (2020) generated the root atlas from 110,427 single cells, by integrating their datasets with the previously published datasets (Denyer et al., 2019; Ryu et al., 2019). Details about the data integrations can be found in Shahan et al. (2020). The large datasets cover all the major root cell-types including QC (1834 cells), columella (11,757), LRC (14,420), atrichoblast (13,972), trichoblast (11,946), cortex (10,318), endodermis (10,541), pericycle (11,603), phloem (6758), xylem (4984), and procambium (12,294). The identity for these cell-types were assigned based on the expression of cell-type-specific marker genes that are well-studied (Shahan et al., 2020).

### Data Analyses and Visualizations

To understand the potential networks of NPK transport systems in Arabidopsis roots, the expression patterns of all the major NPK family channels and transporters were analyzed. Extensive literature searches were performed to identify all the major NPK transporter families in Arabidopsis. The Arabidopsis gene IDs for the NPK transporters were retrieved from The Arabidopsis Information Resource (TAIR^1^). All data were analyzed in R (version 3.5.2) using the batch-corrected (“RNA” assay in Seurat object) expression value. The expression profiles of specific N, P, or K transporter families were plotted using Seurat’s DotPlot function. Unlike the typically used heatmap, the dot plot visualization provides more detailed expression profiling that shows the percentage of cells expressing a specific nutrient channel or transporter gene, and the average expression of cells expressing the gene. This allows identification of the major nutrient transporters that contribute to NPK transport in specific cell type(s) under the normal growth conditions. The differentially expressed Pi transporters in response to low-Pi were extracted from root single cells analyzed by Wendrich et al. (2020) (Supplementary Figure 1). The artworks for potential networks of NPK channels and transporters in the root system were drawn using Adobe illustrator draw application. Nutrient channel and transporter genes expressed under the normal growth conditions were included in the networks.

## Data Availability Statement

The datasets presented in this study can be found in online repositories. The names of the repository/repositories and accession number(s) can be found below: https://www.ncbi.nlm.nih.gov/geo/, GSE152766.

## Author Contributions

DL performed the literature searches, bioinformatic analysis, and wrote the manuscript. SL reviewed and revised the manuscript. Both the authors contributed to the article and approved the submitted version.

## Conflict of Interest

The authors declare that the research was conducted in the absence of any commercial or financial relationships that could be construed as a potential conflict of interest.

## Footnotes

1 https://www.arabidopsis.org/

## Supplementary Material

Supplementary Figure 1 Differentially expressed Pi transporters in specific root cell-types under Pi starvation. The data were retrieved from Wendrich et al. (2020).
