# Supplementary material for: Potential Networks of Nitrogen-Phosphorus-Potassium Channels and Transporters in Arabidopsis Roots at a Single Cell Resolution
Source: Front Plant Sci. 2021 Jun 16;12:689545. doi: 10.3389/fpls.2021.689545 (PMC8242960; doi:10.3389/fpls.2021.689545)
Supplement: Supplementary Figure 1 — Differentially expressed Pi transporters in specific root cell-types under Pi starvation. The data were retrieved from Wendrich et al. (2020). [file Data_Sheet_1.PDF]

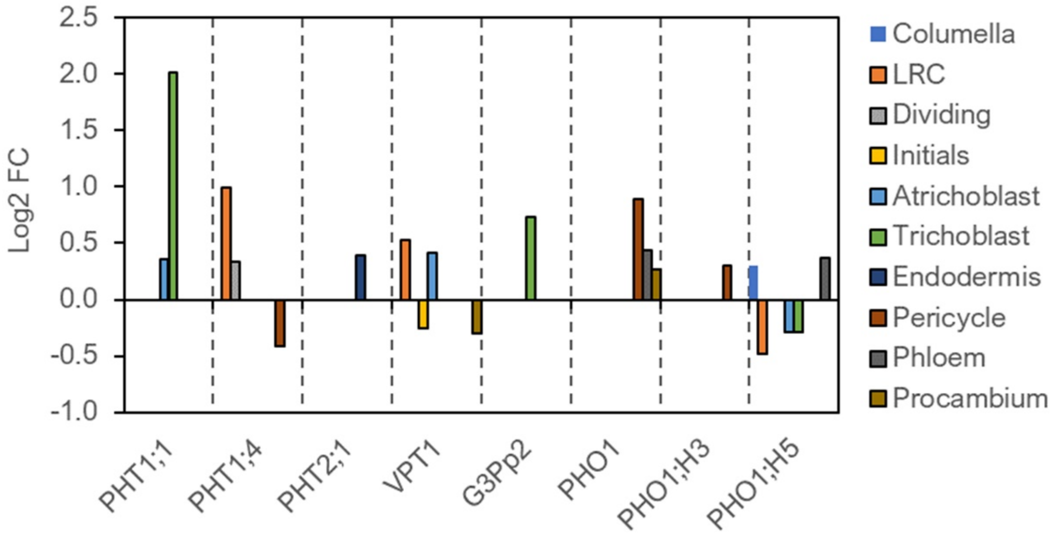

Figure S1 | Differentially expressed Pi transporters in specific root cell types under Pi starvation. The data were retrieved from Wendrich et al., 2020.
